# Increased Upper and Lower Tract Urothelial Carcinoma in Patients with End-Stage Renal Disease: A Nationwide Cohort Study in Taiwan during 1997–2008

**DOI:** 10.1155/2014/149750

**Published:** 2014-06-16

**Authors:** Shuo-Meng Wang, Ming-Nan Lai, Pau-Chung Chen, Yeong-Shiau Pu, Ming-Kuen Lai, Jing-Shiang Hwang, Jung-Der Wang

**Affiliations:** ^1^Department of Urology, National Taiwan University Hospital, Taipei 10002, Taiwan; ^2^Institute of Occupational Medicine and Industrial Hygiene, National Taiwan University, Taipei 10020, Taiwan; ^3^Department of Statistics, Feng Chia University, Taichung 40724, Taiwan; ^4^Chang Gung Memorial Hospital, Taipei 10507, Taiwan; ^5^Institute of Statistical Science, Academia Sinica, Taipei 11529, Taiwan; ^6^Department of Public Health, National Cheng Kung University Medical College, Tainan City 70101, Taiwan; ^7^Departments of Internal Medicine and Occupational and Environmental Medicine, National Cheng Kung University Hospital, Tainan City 70403, Taiwan

## Abstract

*Background*. Urothelial cancer (UC) is the leading cancer of patients with end-stage renal disease (ESRD) in Taiwan. The aims of this study were to explore the time trends of UC incidences and propose possible etiologic factors. *Methods*. Abstracting from the National Health Insurance Research Database (NHIRD), there were 90,477 newly diagnosed cases of ESRD between 1997 and 2008 covering the patients aged 40–85. Among them, 2,708 had developed UC after diagnosis of ESRD. The CIR_40–85_ (cumulative incidence rate) of upper tract UC (UTUC) and lower tract UC (LTUC) were calculated for ESRD patients and general population, as well as SIR_40–85_ (standardized incidence ratio) for comparison. *Results*. Female ESRD patients were found to have 9–18 times of elevated risks of UC, while those of males were increased up to 4–14 times. The time trends of CIR_40–84_ and SIR_40–84_ of UTUC in females appear to decline after calendar year 2000. These trends may be related to AA associated herbal products after 1998. *Conclusions*. Patients with ESRD are at increased risks for both LTUC and UTUC in Taiwan. We hypothesize that the time trends associate with the consumption of aristolochic acid in Chinese herbal products (female predominant).

## 1. Introduction

Various studies carried out in different regions around the world have found that the incidence of malignancy is generally higher in patients suffering from end-stage renal disease (ESRD) [1–4]. Compared with other countries, Taiwan has a remarkably high incidence and prevalence of patients with ESRD [5, 6]. In Taiwan, bladder, liver, and kidney cancers are leading malignancies in patients with chronic dialysis in a 12-year cross-sectional study between 1997 and 2008. Standardized incidence ratios (SIR) of upper tract urinary cancer (UTUC) decline yearly after first dialysis. However, lower urinary tract urothelial cancer (LTUC) persists high in SIR years after first dialysis [7]. However, this study did not explore the possible etiology for the increased risks nor did it stratify for UTUC and renal cell carcinoma.

Among the possible causes for the urothelial cancer, cigarette smoking is among the top candidates associated with increased bladder [8]. Since aristolochic acid (AA) has been documented to be both nephrotoxic and carcinogenic [9, 10], AA-containing CHPs (Chinese herbal products) were banned in November 2003 in Taiwan. However, the effect of banning licensed AA-containing CHPs has never been systematically evaluated.

This study is conducted to determine the cumulative incidence rates (CIR) and SIR in different calendar years for LTUC and UTUC to elucidate the time trends and, furthermore, to preliminarily explore their possible etiologies including tobacco smoking and prescription frequency of AA-containing CHPs. The registry of catastrophic illnesses in Taiwan National Health Insurance and the reimbursement database provides us an excellent opportunity to explore the hypothesis.

## 2. Methods

This study was approved by the Ethics Review Board of the National Taiwan University Hospital before commencement. In addition, the study complies with personal data protection regulations (Data Protection Act).

### 2.1. Study Population

**Figure 1:**
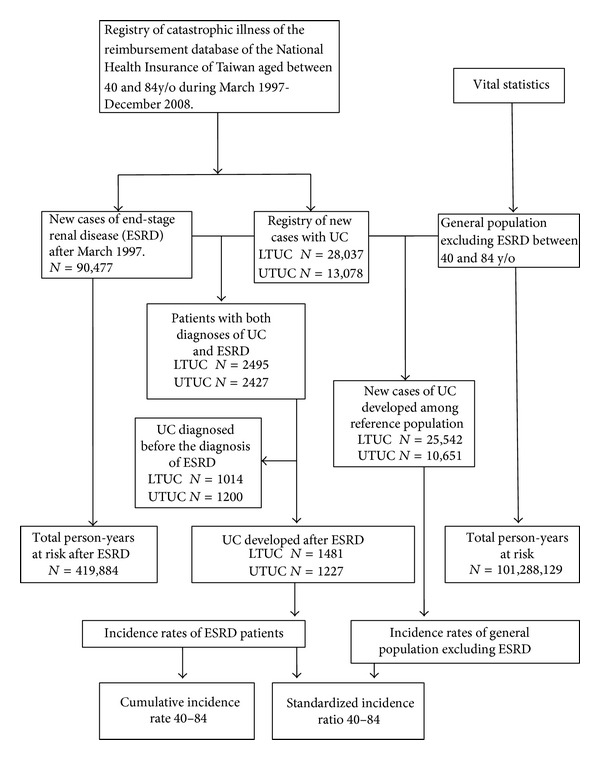
Flowchart of recruitment of subjects in this study. LTUC, lower urinary tract urothelial cancer; UTUC, upper tract urinary tract urothelial cancer; ESRD, end-stage renal disease; CIR, cumulative incidence rate; SIR, standardized incidence ratio.

In this study, the selected subjects registered under catastrophic illnesses from the NHIRD cover the period of 1997–2008. Data on patient demographics (gender and date of birth), diagnoses and treatment (the date treatment began and the date of death or transplantation), and follow-up duration between January 1997 and December 2008 were retrieved. We allowed two months (up to the end of February of 1997) to exclude prevalent cases of ESRD from the above retrieved NHIRD data. Enrolled incident cases included those coded with the following international classification of disease 9th revision (ICD-9) codes: bladder cancer (ICD-9 code 188), renal pelvis and ureter (ICD-9 codes 189.1 and 189.2, resp.), and ESRD (ICD code 585). NHI database enrollees, namely, general population of Taiwan, were taken as the reference group after excluding all the patients with ESRD. Since the age band of urothelial caner (UC) developed cases falls between 40 and 84, we enrolled patients within this category to calculate SIR_40–84_ and CIR_40–84_. The entire procedure for the inclusion of the subjects (aged between 40 and 84) is illustrated in Figure 1.

### 2.2. Statistical Analysis

The study interval is defined from January 1997 to December 31, 2008 since registration system is more comprehensive after early 1997. Follow-up time is calculated as person-year at risk beginning with the date of registered ESRD and ending at situation of death, transplantation, or diagnosis of urinary tract cancer depending on which one comes first. Since all ESRD patients are waived from any copayment in our National Health Insurance (NHI), none (or, extremely rare) would abandon such an important benefit except the deceased. Incidence rates were calculated per 100,000 person-years at risk of UC stratified by age and sex. The age stratified incidence rates for UC in the reference population were used to calculate the number of expected cases under the assumption that the reference population would share the same cancer experience as patients with ESRD for each age stratum. The total number of observed UC cases summed up across all age strata divided by that of expected cases was then defined as the standardized incidence ratio (SIR). We then calculated the 95% confidence intervals (95% CIs) under the assumption of Poisson distribution. The cumulative incidence rate (CIR) formula was calculated as follows: CIR = 1 − exp⁡[−Σ*i*(IR_*i*_)(Δ*t*_*i*_)] where IR_*i*_ represents the age-specific incidence rate and Δ*t*_*i*_ indicates the range of each age stratum. We calculated the CIR_40–84_, which could be interpreted as the cumulative risk of developing UC for an average ESRD patient, if he or she had lived from the age of 40 to 84. All of the above analyses were carried out using the SAS software package, Version 9.1 (SAS Institute Inc., Cary, NC).

We examine the trend of CIR and SIR in every 6-year period from 1998 to 2008 by fitting a simple linear regression model: log⁡⁡(*Y*_*t*_) = *α* + *β* × *t* + *ε*_*t*_, where *t* = 1,2,…, 6 and *Y*_*t*_ is the *t*th CIR or SIR of the examined period [11]. The coefficient *β* determines strength of the trend and the direction of increase or decrease in that period.

## 3. Results

A total of 103,527 newly diagnosed ESRD patients were identified between March 1997 and December 2008. After excluding those who accepted kidney transplantation and those with incomplete patient information, we obtained a total of 90,447 ESRD cases and 419,884 person-years at risk. Within the age group of 40–84, there were approximately 7 million people registered in the NHI system during the period of 1997–2008 (as summarized in Figure 1). After 12 years of follow-up, there were 41,115 UC developed from the general population with an approximate 100 million person-years at risk accumulated. A total of 2,214 cases were excluded from the ESRD cohort because the diagnosis of UC was ahead of that of ESRD, leaving 2,708 new UC cases developed after the diagnosis of ESRD. Development of UTUC and LTUC is persistent after first dialysis. And age is a specific factor in both genders and UTUC and LTUC. About 93.4% of upper tract and 97.4% of lower tract patients were provided with histopathologic reports, which are taken from either biopsy or surgical pathology specimen.

According to the demographic characteristics of ESRD patients, which are summarized in Table 1, there are 1,481 cases of LTUC and 1,227 cases of UTUC enrolled. The SIR_40–84_ of urothelial cancer is summarized in Table 2, which shows an increased trend for all types of UC after 1997 and becomes stabilized after 2004 for both male and female. The female SIRs of different calendar years are generally higher than those of males, of which the SIRs of UTUC are the highest among all UC.

The age-sex specific incidence rates and CIR_40–84_ for all types of UC are summarized in Table 3, which shows that both upper and lower UC occurred in both genders and such trends begin with young age groups. It also shows that the CIR_40–84_ of females are generally higher than those of males in all types of UC for the same time periods with an exception of LTUC in 1997–99. Similar to the SIR_40–84_, CIR_40–84_ of both genders seems to show an increased trend during the 12 years of follow-up and slightly decreased in the last 3 years, especially for UTUC. As the CIR_40–84_ of the general population (excluding ESRD) during the same period have been very stable around 0.021–0.027 in males and 0.007–0.009 in females for LTUC and those of UTUC were between 0.006 and 0.010 in both genders, the epidemic of UC in patients with ESRD (between 0.1 and 0.4 in Table 3) seemed not yet over. Moreover, following inference could be drawn from the SIR_40–84_ in Figure 3: (1) for female UTUC, the increasing trends were found in the first two periods but were not significant. Significant decreasing trend was detected at the last period of 2003–08; (2) female LTUC had significant increasing trends in the first four time periods, but the strength is also weakening. At the last period of 2003–08, the trend disappeared; (3) for male LTUC, significant decreasing trends were found in the periods of 2000–05 and 2001–06; (4) patterns of time trend for male UTUC are similar to those of male LTUC and female UTUC, but no significant trend was found.

## 4. Discussion

Quite different from studies of western countries, we have found that females seem more vulnerable to develop upper or lower location of urothelial cancer after diagnosis of end-stage renal disease, especially in UTUC (Table 1). In fact, female ESRD patients were found to have 9–16 and 11–18 times of elevated risks of LTUC and UTUC, while those of males were increased up to 4–8 and 7–14 times, as summarized in Table 2. The trend of female majority is further corroborated by the estimation of CIR_40–84_ (Table 3). The CIR_40-84_ estimates the probability that in Taiwan an average person with ESRD would develop the event (namely, UTUC or LTUC) if he/she lived up to the age of 85, while the SIR_40–84_ is the total number of observed UC cases summed up across all age strata divided by the total number of expected cases. Moreover, we have found a consistently increasing trend of SIR_40–84_ (Table 2) and CIR_40–84_ (Table 3) of UTUC in ESRD before 2003 and it stabilized or slightly dropped after 2003–05 based on a nationwide data for both males and females. The CIR_40–84_ of LTUC among females were initially lower than those of males before 2000, and they became higher than those of males after 2003 (Table 3). And the SIR_40–84_ of LTUC in males seems to decrease after 2000 but that of females appears elevated throughout the observation period. The CIR_40–84_ of UTUC among females increased about 1.5 times that of males before 2000, which further climbed up to about 2 times.

It is interesting to know when the UC develops after initiation of dialysis. SIR of UTUC increased up to about more than 20–30 times in the first year after dialysis and then decreased slowly during the 12 years of observation [7]. The decreasing trend along calendar period could be expressed in the regression coefficient of Figure 3, especially among the females. However, while the male LTUC seems to show a decreasing trend expressed by the consistent negative signs of linear regression coefficients, those of females remain elevated in SIR until the period of 2003–8 to show a negative sign. We have also summarized the cumulative incidence rates of developing UC along time after dialysis in Figure 4.

Given such increased risks, we must consider the potential etiological factors. In general, risk factors that are reported to be associated with urothelial cancer include analgesics (phenacetin), herbal usage (aristolochic acid), heavy metals (arsenic), and tobacco smoking reported in international agency in research on cancer (IARC) [12]. Long-term use of phenacetin or NSAID is a potential risk factor for LTUC [13, 14]. Since IARC classified phenacetin as category 1 carcinogen for urothelial cancer in 1987, its compounds have been banned from Taiwan market since July of 1985 [15]. Moreover, patients with ESRD under dialysis are instructed to avoid taking NSAID (nonsteroidal anti-inflammatory drugs), which were illustrated by our data showing 99% of ESRD patients were prescribed NSAID for less than 500 pills within the two years before and after dialysis in both cancer and reference groups. Thus, phenacetin and/or NSAIDs did not seem to explain the above increased trend for both UTUC and LTUC. Since all patients with ESRD are recommended to avoid any food or medication suspected to contain metals that might accumulate in our body and only one out of 2708 cases of urothelial cancer after ESRD resided in the area of endemic arsenic poisoning (blackfoot disease), the etiological connection with arsenic seems unlikely. A preliminary study determining both arsenic and cadmium content in renal parenchyma tissue of three UTC cancers among patients with ESRD did not find any increase of these two metals in comparison with 26 samples without such cancers.

In several studies smoking is strongly related to LTUC and possibly UTUC in occurrence, recurrence, and progression [8, 12, 16]. Prevalence rate of tobacco consumption appears decreased from 60% down to below 40% in males, while that in females has remained about 5% or lower during the last 20 years according to data from the Department of Health, Executive Yuan in a survey of general population [17]. Although the whole picture appears compatible with the trend of decreased LTUC in males (Figure 3), it cannot explain the increased SIR_40–84_ in females after age standardization. Moreover, almost all patients with ESRD were usually advised to quit or stay away from smoking by physicians. Since the time trends of UTUC for both genders in Table 2 were unrelated to the trends of cigarette consumption in Taiwan, we thus concluded that the increased UTUC in ESRD patients is probably not associated with smoking.

**Figure 2:**
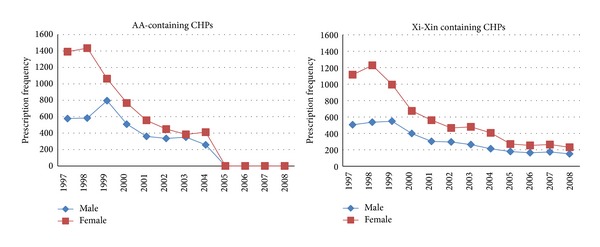
Prescription frequencies of aristolochic acid (AA) related and Xi-Xin Chinese herbal products (CHPs) in 90,477 patients with ESRD (end-stage renal disease), stratified by sex.

**Figure 3:**
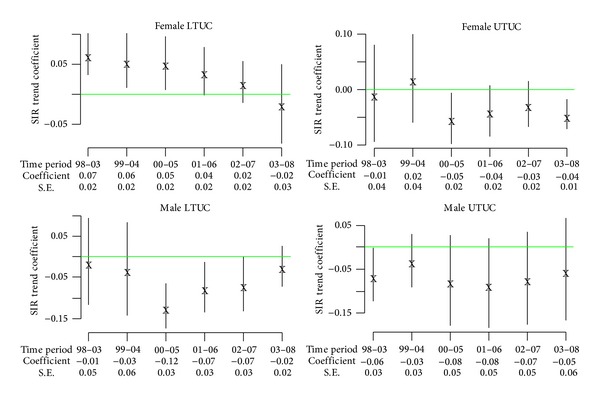
Estimated coefficients with 95% confidence intervals and standard errors (S.E.) for SIRs (standardized incidence ratios) linear trend during specified periods.

**Figure 4:**
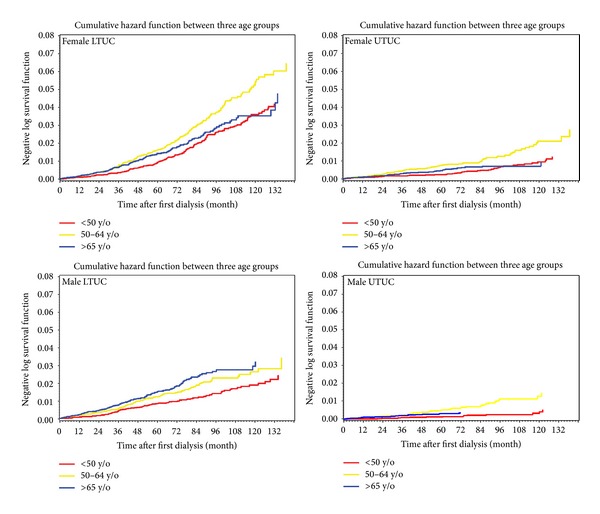
Accumulative hazard function to demonstrate the incidence rates of developing UC (urothelial carcinoma) along time after first dialysis stratified by sex, age (<50, 50–64, >65 years old), and upper tract (UTUC) and lower tract (LTUC).

Aristolochic acid has been documented as nephrotoxic and carcinogenic and its related products or remedies are banned in many countries, including USA, UK, and Canada [18]. The above trends appear to correspond with the decreased consumption of several AA-containing products beginning in 1999 (Figure 2) and the ban on AA-containing CHPs in Taiwan on November 3 of 2003 (Figure 2) [8, 18]. The time trends of CIR_40–84_ of LTUC and UTUC in females appear to decline after calendar year 2000. This trend seems compatible with reduced consumption after 1998 and the ban of aristolochic acid (AA) associated herbal products in 2003. After the year of 2000, the SIR_40–84_ of UTUC appears to drop in both genders and the SIR_40–84_ of LTUC declines in male significantly. These trends may be related to decreased consumption of cigarettes in males after 1990 and AA associated herbal products after 1998. Similar to what was reported in the AA related cancer in Belgium, the peak of LTUC usually appears as early as 3-4 years later than that of UTUC [19, 20]. Unfortunately, CHP that contains Xi-Xin is still available in market of Taiwan [21, 22], which might contain minute amount of AA after 25 times of concentration of the products and might still produce carcinogenic effect for patients with ESRD whose kidney is unable to excrete toxics [23]. As Xi-Xin is totally banned in America and Europe, AA related DNA adduct has been documented in cases of UTUC in Taiwan [24, 25]. We are currently conducting another study to estimate the doses of AA from prescribed Chinese herbal products and the association with occurrence of UC in dialysis patients, which may provide a more direct evidence for Taiwan to consider a similar action.

Limitations of this study include possible ecological fallacy and the lack of personal genetic and life style information from the NHRI database, and details of tumor staging are not available in the NHIRD. We are also unable to quantify the exact consumption of analgesics and CHPs, as many of them are sold without prescription. Since the results of this study provide a preliminary inspection of the trend, they must be interpreted cautiously, and more studies are needed in the future to corroborate the proposed hypothesis.

## 5. Conclusions

Patients with ESRD requiring dialysis have increased after beginning of NHI system in Taiwan since 1995. Female patients diagnosed of ESRD were found to have 9–16 and 11–18 times of elevated risks of LTUC and UTUC, while those of males were increased up to 4–8 and 7–14 times. The time trends of SIR_40–84_ and CIR_40–84_ of UTUC seem to synchronize with the increased prescription and consumption of AA associated herbs until 2000 [8, 26]. Further corroboration of this hypothesis may uncover the actual etiology and prevent more victims from urothelial cancer in Taiwan.

## Supplementary Material

Estimated trend coefficients with 95% confidence intervals for CIR's (cumulative incidence rates). This figure shows that for male LTUC, significant decreasing trends were found in all the 6-year periods after 1999. There were decreasing trends for male UTUC after 2000 and female LTUC and UTUC after 2003, but none of them show a statistical significant trend.

## Figures and Tables

**Table 1:** Characteristics of patients with end-stage renal disease between 40 and 84 years old under maintenance dialysis in the registry of catastrophic illnesses between March 1997 and December 2008.

	Male	Female
Number of the subjects with ESRD	43,937	46,496
Total person-years at risk	197,753	222,131
Age at start of dialysis (total person-years)		
<40 years	35,307	26,653
40–49 years	35,821	41,327
50–59 years	50,873	50,373
60–69 years	56,140	66,570
70–84 years	54,919	63,861
Duration of follow-up years: number (%)		
<1 year	10,575 (24.1)	10,313 (22.1)
≥1 and <5 years	19,426 (44.2)	19,701 (42.4)
≥5 and <10 years	11,543 (26.3)	13,415 (28.9)
≥10 years	2393 (5.4)	3067 (6.6)
Urothelial cancer cases		
Lower tract urothelial cancer: number (%)	634 (42.8)	847 (57.2)
Upper tract urothelial cancer: number (%)	417 (34.0)	810 (66.0)

**Table 2:** Age-standardized incidence rates (ages between 40 and 84) for patients under maintenance dialysis stratified by sex and calendar year.

	LTUC		UTUC	
	O/E	SIR_40–84_ (95 %CI)	O/E	SIR_40–84_ (95 %CI)
Male				
1998	16/2.7	5.9 (3.6–9.7)	7/0.5	13.0 (6.2–27.3)
1999	27/6.0	4.5 (3.0–6.6)	18/1.3	13.9 (8.8–22.1)
2000	48/5.7	8.4 (6.4–11.2)	19/1.6	12.2 (7.8–19.1)
2001	43/6.8	6.3 (4.7–8.5)	28/2.2	12.9 (8.9–18.7)
2002	68/8.4	8.1 (6.4–10.3)	34/2.9	11.9 (8.5–16.6)
2003	65/10.5	6.2 (4.8–7.9)	49/3.5	13.9 (10.5–18.4)
2004	63/12.5	5.1 (4.0–6.5)	53/4.1	12.8 (9.8–16.8)
2005	61/13.7	4.5 (3.5–5.8)	35/4.9	7.2 (5.2–10.0)
2006	74/14.0	5.3 (4.2–6.6)	52/5.5	9.4 (7.2–12.3)
2007	79/14.7	5.4 (4.3–6.7)	58/6.1	9.5 (7.3–12.3)
2008	83/16.6	5.0 (4.0–6.2)	65/5.8	11.2 (8.8–14.2)
Female				
1998	12/1.3	9.0 (5.1–15.8)	11/0.8	14.3 (7.9–25.8)
1999	29/3.1	9.5 (6.6–13.7)	20/1.8	10.9 (7.1–16.9)
2000	36/3.3	10.8 (7.8–14.9)	47/2.7	17.7 (13.3–23.6)
2001	49/3.9	12.6 (9.5–16.6)	56/3.1	18.4 (14.1–23.9)
2002	76/5.1	14.8 (11.8–18.5)	57/4.1	13.9 (10.7–18.0)
2003	83/5.5	15.1 (12.1–18.7)	83/5.0	16.5 (13.3–20.4)
2004	89/6.7	13.3 (10.8–16.4)	94/5.7	16.6 (13.5–20.3)
2005	118/7.3	16.1 (13.5–19.3)	93/6.8	13.7 (11.2–16.8)
2006	136/8.3	16.4 (13.9–19.5)	113/7.8	14.5 (12.1–17.4)
2007	121/7.4	16.3 (13.7–19.5)	107/7.9	13.5 (11.2–16.3)
2008	100/8.3	12.0 (9.9–14.6)	116/8.5	13.6 (11.4–16.4)

LTUC: lower urinary tract urothelial cancer; UTUC: upper urinary tract urothelial cancer; and SIR: standard incidence rate.

**Table 3:** Sex- and age-specific rates (per 100,000 person-years) and cumulative incidence rates up to 84 years old (CIR_40–84_) of urothelial cancer calculated for every 3-year interval between 1997 and 2008.

	Age category	Male		Female	
		LTUC	UTUC	LTUC	UTUC
1997–99	40–49	372.0	148.8	361.9	180.9
	50–59	491.3	109.2	600.0	250.0
	60–69	774.9	122.3	771.1	175.3
	70–84	952.8	129.9	504.5	232.8
	CIR_40–84_	0.3	0.1	0.2	0.1

2000–02	40–49	400.1	100.0	700.1	140.0
	50–59	1013.8	362.1	999.5	309.4
	60–69	1027.3	296.3	1059.3	554.1
	70–84	1103.5	120.4	948.5	210.8
	CIR_40–84_	0.3	0.1	0.3	0.1

2003–05	40–49	327.9	131.2	526.6	292.6
	50–59	832.1	282.3	1125.7	616.8
	60–69	701.8	259.9	1158.0	496.3
	70–84	1035.8	172.6	1140.5	245.2
	CIR_40–84_	0.3	0.1	0.4	0.2

2006–08	40–49	274.7	85.9	511.0	148.4
	50–59	639.4	108.4	1366.9	431.7
	60–69	810.8	170.7	1018.6	392.5
	70–84	840.4	166.1	992.6	215.1
	CIR_40–84_	0.3	0.1	0.4	0.1

LTUC: lower urinary tract urothelial cancer; UTUC: upper urinary tract urothelial cancer; and CIR: cumulative incidence rate.
