# Supplementary material for: Increased Upper and Lower Tract Urothelial Carcinoma in Patients with End-Stage Renal Disease: A Nationwide Cohort Study in Taiwan during 1997–2008
Source: Biomed Res Int. 2014 Jun 16;2014:149750. doi: 10.1155/2014/149750 (PMC4084494; doi:10.1155/2014/149750)
Supplement: Supplementary file 1 — Estimated trend coefficients with 95% confidence intervals for CIR's (cumulative incidence rates). This figure shows that for male LTUC, significant decreasing trends were found in all the 6-year periods after 1999. There were decreasing trends for male UTUC after 2000 and female LTUC and UTUC after 2003, but none of them show a statistical significant trend. [file 149750.f1.docx]

Figure. Estimated trend coefficients with 95% confidence intervals for CIR’s (cumulative incidence ratios)
